# The relationship between common mental disorders and incident diabetes among participants in the Kerala Diabetes Prevention Program (K-DPP)

**DOI:** 10.1371/journal.pone.0255217

**Published:** 2021-07-23

**Authors:** Leslie C. M. Johnson, Allissa Desloge, Thirunavukkarasu Sathish, Emily D. Williams, Pilvikki Absetz, Tilahun Haregu, Jeroen De Man, Kavumpurathu Raman Thankappan, Brian Oldenburg

**Affiliations:** 1 Department of Family and Preventive Medicine, School of Medicine, Emory University, Atlanta, Georgia, United States of America; 2 Hubert Department of Global Health, Rollins School of Public Health, Emory University, Atlanta, Georgia, United States of America; 3 School of Population and Global Health, University of Melbourne, Melbourne, Australia; 4 MacMillan Center for International and Area Studies, Yale University, New Haven, Connecticut, United States of America; 5 Population Health Research Institute, McMaster University, Hamilton, Ontario, Canada; 6 School of Health Sciences, University of Surrey, Guildford, United Kingdom; 7 Collaborative Care Systems Finland, Tampere University, Tampere, Finland; 8 Centre for General Practice, Department of Primary and Interdisciplinary Care, University of Antwerp, Antwerp, Belgium; 9 Department of Public Health and Community Medicine, Central University of Kerala, Kasaragod, India; Shanghai Jiao Tong University, CHINA

## Abstract

This study aims to describe the prevalence of depression and anxiety among a population sample of people at high risk for type 2 diabetes in Kerala, India, and examine the relationship between depressive symptoms, anxiety, and incident Type 2 Diabetes Mellitus (T2DM) over a two-year period. We used data from the Kerala Diabetes Prevention Program, a cluster-randomized controlled trial for diabetes prevention among 1007 high-risk individuals. The prevalence of depression and anxiety were estimated using the 9-item Patient Health Questionnaire and the Generalized Anxiety Disorder 7-item scale, respectively. We calculated proportions for depression and anxiety and performed generalized estimating equations (GEE) to examine the relationship between baseline mental health status and incident T2DM. The prevalence of depression and anxiety at baseline were 7.5% and 5.5%, respectively. Compared with those reporting none/low symptoms, the odds ratio for incident diabetes was 1.07 (95% CI 0.54–2.12) for participants with moderate to severe depression and 0.73 (95% CI 0.23–2.28) for participants with moderate to severe anxiety, after adjusting for potential confounders. Our findings suggest that the prevalence of depression and anxiety were higher than those previously reported in the general population in India. However, among this sample of community-based adults at high risk of developing T2DM, the presence of moderate to severe depression and/or anxiety symptoms was not significantly associated with the risk of developing T2DM.

**Trial registration**: Australia and New Zealand Clinical Trials Registry ACTRN12611000262909. Registered 10 March 2011.

## Introduction

Addressing common mental disorders (CMDs), such as depression and anxiety, has become a global health priority; CMDs account for around a third of non-fatal disease burden worldwide and around 10 percent of overall disease burden [1]. CMDs can have a very detrimental impact on an individual’s well-being and often co-occur with other chronic conditions [2] such as type 2 diabetes mellitus (T2DM) and cardiovascular diseases. The cumulative impact of these comorbidities can lead to worsening health outcomes, decreased quality of life, and poor chronic disease self-management.

There is now a significant body of research examining the relationship between diabetes and CMDs. In particular, there is robust evidence indicative of a bidirectional relationship between diabetes and depression in population-based samples or among people with T2DM [3, 4]. However, findings are mixed on whether depression significantly increases the risk of T2DM. A meta-analysis examining the relationship between depression and incident diabetes found that 8 of 15 studies demonstrated an increased risk of T2DM among those with depression [3]. These studies found a 1.41 fold increase in risk or 1.24 fold increase in hazard for T2DM among adults with depression [3]. Notably, of 32 studies conducted from 1980–2016 examining the risk of diabetes in adults with depression, only two were conducted in low and middle-income countries (LMICs); with the highest documented risk for diabetes among people with depression occurring in South Asia [3–5].

While there are far fewer studies examining the relationship between anxiety and T2DM, of which the existing evidence is inconsistent, there is also some evidence that anxiety has been shown to be a predictor for incident T2DM. A meta-analysis found that 10 of 14 studies reported significant associations between baseline anxiety and incident diabetes [6]. Among these studies, none have been conducted in LMICs. Given that the evidence on such comorbidities is largely derived from studies conducted in high-income counties, it is important to examine the relationship between diabetes and CMDs in LMICs.

Current projections estimate that by 2030 developing countries will see a 69% increase in the number of people with diabetes, compared to a 20% increase in developed countries, with India having the largest absolute number of adults with diabetes in the world [7]. Diabetes affects approximately 77 million adults in India [8]. As the prevalence of diabetes in India, currently 8.9% [8], continues to increase, a similar trend is also occurring for mental health disorders. According to the 2016 National Mental Health Survey of India, the overall lifetime prevalence for any mental health condition among adults is 13.7% [9]. Prevalence rates in India for both diabetes and mental health conditions vary depending on the region/state. Diabetes prevalence in the state of Kerala is around 19.2% [10], while the prevalence for depression and generalized anxiety disorder are around 2.7% and 0.6%, respectively [9]. The World Health Organization (WHO), however, estimates the prevalence of depressive disorders to be 4.5% and anxiety disorders to be 3% in the South East Asia region [1]. These data highlight the need to find effective methods of both preventing and treating diabetes and mental disorders in LMICs and India, in particular.

To date, however, the majority of research has centered on documenting the burden of mental disorders among people with diabetes and testing depression and diabetes treatment models. A recent systematic review found a significant relationship between diabetes and depression among Indian populations with diabetes, with a pooled prevalence of depression about 38% [11]. Anxiety affects up to 34% of people with diabetes in India [12], while the prevalence of depression has been documented at 49% of people with diabetes in Kerala [13]. One treatment approach, collaborative care, has been found effective at improving diabetes outcomes through the treatment of co-morbid mental disorders [14–17]. This model of care originates from high-income countries, but was recently adapted and tested in India. Ali and colleagues [18] found that, among patients with depression and diabetes who were engaged in a collaborative care intervention, co-treating these comorbidities resulted in lower depressive symptoms and improved cardiometabolic indices. Since considerable research has focused on the mental health of people with diabetes and how the comorbidity between diabetes and depression leads to poorer diabetes related outcomes, further research is needed focusing on the relationships between depression, anxiety, and T2DM among people who are at risk for developing diabetes, and for whom intervention may prevent the development of these comorbidities. To our knowledge, the present study is the first to examine the relationship between diabetes and CMDs in Indian populations at risk for developing diabetes.

In this study, we aimed to 1) describe the prevalence of depression and anxiety among a population sample of people at high risk for type 2 diabetes in Kerala and 2) examine the relationship between depressive symptoms, anxiety, and incident T2DM over a two-year follow-up period; adjusting for sociodemographic, behavioral, and clinical factors associated with these chronic conditions [3, 6]. Sociodemographic risk factors for diabetes include age, sex, education, marital status, and family history of T2DM [19, 20]. Behavioral risk factors for diabetes include alcohol use, tobacco use, physical activity and diet [21]. Clinical risk factors for diabetes include BMI, central obesity, cholesterol levels, and blood pressure [22]. Several of these risk factors are also shared with CMDs, including age, sex, education, marital status, BMI, and poor lifestyle factors [23–28].

## Participants and methods

The study was approved by the Institutional Ethics Committee of the Sree Chitra Tirunal Institute for Medical Sciences and Technology, Trivandrum, Kerala, and by the Human Research Ethics Committees of Monash University, Australia and the University of Melbourne, Australia. The study was also approved by the Health Ministry Screening Committee of the Government of India, meeting all national level requirements to conduct this research. Written informed consent was obtained from all study participants.

### Study design and participants

This study included participants from a cluster-randomised controlled trial, the Kerala Diabetes Prevention Program (K-DPP). A detailed description of the K-DPP study design and methods [29] has been previously been published. Briefly, a total of 2,586 adults were screened in 2013 from 60 polling areas (clusters) in the Trivandrum district of Kerala for participation in the K-DPP. Eligible participants included individuals aged between 30 and 60 years, with no history of T2DM, chronic illnesses, taking glucose lowering medications, who are not pregnant, and who scored ≥60 on the Indian Diabetes Risk Score (IDRS), indicating that they are at high-risk of developing T2DM (20). Those diagnosed with T2DM (fasting plasma glucose (FPG) ≥7.0 mmol/ and/or 2-hr plasma glucose (2-hr PG) ≥11.1 mmol/l [30]) on a 2-hr oral glucose tolerance test were excluded, and referred to health facilities for treatment and care. The remaining individuals with prediabetes or normoglycemia were included in the trial. A total of 1,007 individuals were included in the K-DPP randomised trial, testing the effectiveness of a peer-support lifestyle program [31] designed to prevent or delay the onset of T2DM at two years. Participants who had complete data for diabetes incidence (n = 919) were included in the aim 2 analyses as a cohort.

### Measures

Data on IDRS, socio-demographic characteristics, and anthropometrics were collected during home visits. All other measures were obtained at community-based clinics at baseline, one and two years (see data in S1 Dataset and the data analysis codes in S2 Dataset). Questionnaires were administered by trained interviewers. These tools were translated to the local language.

#### Depression and anxiety

The nine-item Patient Health Questionnaire (PHQ-9), a valid and reliable depression severity measure [32], was administered at baseline to assess the presence and severity of depression among participants. Each item is based on depression criteria from the Diagnostic and Statistical Manual of Mental Disorders and is scored from 0 (not at all) to 3 (nearly every day). The PHQ-9 total summative score ranges from 0 to 27. A cut-off score of 10 was used to classify people with moderate to severe depression (sensitivity (82.2%) and specificity (84.7%) are maximized compared to other cut-points while maintaining acceptable diagnostic properties for detecting major depressive disorder) [33, 34]. The PHQ-9 demonstrated good internal consistency with this sample (Cronbach’s α = 0.80).

The seven-item Generalized Anxiety Disorder Scale (GAD-7) was administered at baseline to assess the presence and severity of generalized anxiety disorder among participants. The GAD-7 was developed and validated within a primary care setting [35], and has since been validated in community settings [36]. Response options for each item range from 0 (not at all) to 3 (nearly every day), with a total score range of 0 to 21. A cut-off score of 10 was used to classify people with moderate to severe anxiety status as this cut-off has been shown to have good sensitivity (89%) and specificity (82%) [35]. The GAD-7 demonstrated good internal consistency with this sample (Cronbach’s α = 0.84).

A psychometric validation of the translated PHQ-9 and GAD-7 instruments supports the use of both scales to measure anxiety and depressive symptom severity among individuals in India [37].

#### Sociodemographic measures

Data on age, sex, years of education, marital status, and family history of diabetes were collected by interviewer-administered standardized questionnaires. Age and years of education were reported as continuous values, while sex (male or female) and family history of diabetes (yes or no) were dichotomized response options. Marital status was a categorical variable with five response options (i.e., married, separated, divorced, widowed, single) that were combined to create two response options: married or unmarried.

#### Behavioral measures

Alcohol use was assessed with the item: “Did you consume an alcoholic drink (beer, wine, whiskey, toddy (local alcoholic drink)) in the last 30 days?” (yes/ no) [38]. Tobacco use was assessed with the item: “Did you use any of the following tobacco products (smoking: cigarettes, bidis, cigars, hookah; smokeless: snuff, betel with tobacco, khaini, gutka) in the last 30 days?” Participants were categorized as using tobacco if they reported using smoking tobacco, smokeless tobacco, or both in the last 30 days. Fruit and vegetable intake were assessed through an adapted Food Frequency Questionnaire (FFQ) [10]. Participants were classified as those who consumed <5 or ≥5 servings of fruits and vegetables per day, in accordance with the WHO STEPs manual [38]. Leisure time physical activity was defined as self-reported history of moderate or vigorous physical activities during leisure time performed in bouts of at least 10 minutes duration on a typical day in a usual week, based on questions from the Global Physical Activity Questionnaire (GPAQ) [39].

#### Clinical risk factor measures

Body mass index (BMI) was calculated using clinical measures of height, collected using a Seca stadiometer, and weight, using a TANITA body composition analyzer, with the formula: weight (kg) / height (m^2^). BMI was categorized as underweight (<18.5 kg/m^2^), normal (18.5–22.9 kg/m^2^), overweight (≥23 but <25 kg/m^2^), and obese (≥25 kg/m^2^) [40]. Central obesity was defined as waist circumference ≥90 cm in men and ≥80 cm in women [41], measured using a Seca measuring tape in accordance with the guidelines provided in the WHO STEPS manual [38]. Blood pressure readings were taken using an Omron automatic blood pressure monitor. The average of the second and third of three readings was utilized to create systolic blood pressure (SBP) and diastolic blood pressure (DBP) variables. Hypertension was then defined as SBP≥140 mmHg and/or DBP≥90 mmHg and/or currently taking blood pressure lowering medication [42]. Blood samples were taken from each participant to provide measurements for plasma glucose levels, serum lipids, and glycated haemoglobin (HbA1c) in mmol/mol and as a percentage (Diabetes Control and Complication Trial units). LDL cholesterol (mmol/l) was utilized for this analysis because it is considered to be the primary predictor of metabolic conditions and the most important therapeutic target for reducing coronary risk among people with diabetes [43].

#### Diabetes outcome variable

In accordance with the American Diabetes Association criteria, we categorized participants as having incident T2DM over two years if they had a FPG of ≥ 7.0 mmol/l and/or 2-hr PG of ≥11.1 mmol/l and/or clinically diagnosed by a physician and taking glucose lowering medications [30].

### Statistical analysis

Data are summarized using frequency and percentage for categorical variables, and by mean (SD, standard deviation) for continuous variables. Since we are interested in estimating population-average effects (or marginal inference), we used Generalized Estimating Equations (GEE) [44] to model the associations between depression and anxiety with incident diabetes with the *xtg*ee command in Stata. GEE only requires correct specification of the distribution of the dependent variable so that the variance can be efficiently calculated as a function of the mean and the regression coefficients can be properly interpreted. Since our dependent variable (i.e., incident diabetes) was binary, we specified binomial family with ‘logit’ link function to connect the covariates and marginal means, as recommended by McCullagh and colleagues [45]. An exchangeable correlation structure was specified, as it is likely that observations of the dependent variable over time would have the same correlation (estimated correlation was 0.01) [46, 47]. Further, GEE can provide valid coefficients even when the working matrix is misspecified [44, 48]. Standard errors were based on Huber-White sandwich estimator (or robust variance estimator), which provides valid confidence intervals (CIs) even if the correlation structure is misspecified [44]. Results of GEE models (univariate and multivariable) are presented as odds ratios, 95% CIs, and p values [49–51].

Covariates (or confounders) were chosen from the literature and based on their possible associations with incident diabetes [49–51]. The correlation was stronger between central obesity and T2DM compared to obesity based on BMI among K-DPP participants (34), therefore we considered central obesity as the measure of adiposity in the analyses. We modelled the associations between depression and anxiety with incident diabetes, after adjustments for baseline covariates in the following hierarchical manner:

Model 1: adjusted for study arm, sex, age, years in school, marital status, and family history of diabetes.
Model 2: adjusted for study arm, sex, age, years in school, marital status, and family history of diabetes, alcohol use, tobacco use, leisure time physical activity, and fruit and vegetable consumption.
Model 3: adjusted for study arm, sex, age, years in school, marital status, and family history of diabetes, alcohol use, tobacco use, leisure time physical activity, fruit and vegetable consumption, central obesity, hypertension, and LDL cholesterol.

The goodness-of-fit of each model was assessed using Pan’s Quasilikelihood under the Independence model Criterion (QIC), with the model having the lowest QIC value deemed to have the best fit [52].

To examine the possibility of participation bias, we compared individuals who had ≥1 follow-up visits (n = 958) and those that did not attend any follow-up visit (n = 49) using descriptive statistics. No hypothesis testing technique was used for this purpose, as there is a high likelihood for a type II error, given the small number of participants with no follow-up visits.

Missing data occurred in 8.7% of the diabetes incidence data, in 3.6% of the PHQ-9 data, 0.4% of the GAD-7 data, and 0.5% of the central obesity data. All other variables included in the GEE models were 100% complete. To assess the robustness of our main results (complete case analyses) to missing data, we performed 10 multiple imputations (MI) using chained equations [53]. GEE models were run on each of the 10 MI datasets (see data in S3 Dataset), and the results were combined using Rubin’s rule [54]. A two-sided P <0.05 indicates statistical significance. All statistical analyses were performed using the Stata/SE 15.1 software package.

### Ethics approval

The study was approved by the Institutional Ethics Committee of the Sree Chitra Tirunal Institute for Medical Sciences and Technology, Trivandrum, Kerala, and by the Human Research Ethics Committees of Monash University, Australia and the University of Melbourne, Australia. The study was also approved by the Health Ministry Screening Committee of the Government of India, meeting all national level requirements to conduct this research.

## Results

Of the 1,007 individuals enrolled in the K-DPP, 919 (91.2%) had complete incident T2DM data over two years. A detailed description of baseline characteristics of the K-PP participants has been previously reported [55].

### Aim 1

At baseline, 7.5% and 5.5% of participants scored > = 10 on the PHQ-9 and GAD-7, respectively. Of those who completed both mental health questionnaires, 2.7% had > = 10 on both the PHQ-9 and GAD-7.

### Aim 2

Table 1 illustrates the comparison of all the measures across those that developed T2DM compared with those that did not over the two-year follow-up period. Compared with participants who did not develop diabetes, individuals that developed T2DM had greater baseline adiposity (central obesity and BMI), as well as more baseline hypertension, and higher levels of LDL (all p<0.05).

**Table 1:** Baseline characteristics of K-DPP participants according to incident diabetes at 2 years.

Characteristics	Incident Diabetes		Odds Ratio (95% CI)	P value
	Yes (*N* = 147)	No (*N* = 772)		
Age, in years; M (SD)	46.3 (7.1)	46.0 (7.5)	1.01 (0.99, 1.04)	0.29
Sex, female; N (%)	66 (44.9)	378 (49.0)	0.81 (0.55, 1.20)	0.29
Marital status, married; N (%)	136 (92.5)	739 (95.7)	0.66 (0.32, 1.35)	0.25
Education, years completed; M (SD)	10.1 (3.9)	9.6 (3.8)	1.02 (0.98, 1.07)	0.34
Family history of diabetes, Yes; *N* (%)	75 (51.0)	402 (52.1)	1.02 (0.75, 1.39)	0.89
Alcohol use, Yes; N (%)	32 (21.8)	156 (20.2)	1.16 (0.81, 1.68)	0.42
Tobacco use, Yes; N (%)	35 (23.8)	141 (18.3)	1.43 (1.00, 2.04)	0.05
Fruit and vegetable intake, <5 servings per day; N (%)	96 (65.3)	540 (70.0)	0.90 (0.64, 1.27)	0.56
Leisure time physical activity, No; N (%)	122 (83.0)	614 (79.5)	0.73 (0.47, 1.12)	0.15
Central obesity, Yes; N (%)	140 (95.9)	712 (92.7)	1.51 (1.06, 2.15)	0.024
Hypertension, Yes; N (%)	45 (30.6)	160 (20.7)	1.66 (1.08, 2.54)	0.021
LDL cholesterol, in mg/dl; M (SD)	154.2 (35.0)	146.3 (35.7)	1.01 (1.00, 1.01)	0.013
PHQ-9, N (%)				
<10	130 (92.9)	691 (92.8)	Ref	
≥10	10 (7.1)	54 (7.3)	1.00 (0.53, 1.88)	1.00
GAD-7, N (%)				
<10	142 (96.6)	725 (94.4)	Ref	
≥10	5 (3.4)	43 (5.6)	0.72 (0.24, 2.16)	0.56

M, mean; SD, standard deviation; LDL, low density lipoprotein; PHQ, Patient Health Questionnaire; GAD-7, Generalized Anxiety Disorder-7. Percentages may not add up to 100% because of rounding. Comparison of characteristics between those with incident diabetes and those without was performed using Generalized Estimating Equations with an exchangeable working matrix. Binomial family and logit link function were specified in the models. Standard errors were based on Huber-White sandwich estimator.

Table 2 shows multivariable analyses with the development of T2DM as the dependent variable. Diabetes developed in approximately 16% of the trial participants who were followed-up at two years.

**Table 2:** Association of depression and anxiety with incident diabetes in multivariable analysis.

Model	Risk Factor	Odds Ratio	95% CI	P-value	QIC
Model 1^1^	PHQ-9 Score ≥10	1.07	(0.55, 2.08)	0.85	1563.71
	GAD-7 Score ≥10	0.73	(0.24, 2.25)	0.59	1635.04
Model 2^2^	PHQ-9 Score ≥10	1.02	(0.51, 2.01)	0.96	1566.82
	GAD-7 Score ≥10	0.72	(0.24, 2.17)	0.56	1641.87
Model 3^3^	PHQ-9 Score ≥10	1.07	(0.54, 2.12)	0.85	1539.35
	GAD-7 Score ≥10	0.73	(0.23, 2.28)	0.58	1615.45

PHQ, Patient Health Questionnaire; GAD-7, Generalized Anxiety Disorder-7; CI, confidence interval; QIC, Quasilikelihood under the Independence model Criterion.

Odds ratios (and 95% CIs) were estimated using Generalized Estimating Equations with an exchangeable working matrix. Binomial family and logit link function were specified in the models. Standard errors were based on Huber-White sandwich estimator.

^1^Model 1: adjusted for study arm, sex, age, years in school, marital status, and family history of diabetes.

^2^Model 2: adjusted for study arm, sex, age, years in school, marital status, and family history of diabetes, alcohol use, tobacco use, leisure time physical activity, and fruit and vegetable consumption.

^3^Model 3: adjusted for study arm, sex, age, years in school, marital status, and family history of diabetes, alcohol use, tobacco use, leisure time physical activity, fruit and vegetable consumption, central obesity, hypertension, and LDL cholesterol.

Model 3 had the best fit for both depression and anxiety as it had the lowest QIC values. Compared with those reporting none/low symptoms, the odds ratio for incident diabetes was 1.07 (95% CI 0.54–2.12) for participants with moderate to severe depression and 0.73 (95% CI 0.23–2.28) for participants with moderate to severe anxiety after adjusting for potential confounders. The results of these complete case analyses were similar to those from the multiple imputations MI analyses (see S1 Table for results of the multivariable analysis on MI datasets). Participants lost to follow-up were more likely to be men, have hypertension and central obesity, and reported more alcohol and tobacco use, less leisure time physical activity than participants who were not lost to follow-up (see S2 Table).

## Discussion

This study aimed to understand the relationship between CMDs and the development of T2DM among individuals who were at risk of developing diabetes in India. The prevalence of depression in our study population was more than double that previously reported in Kerala, and the prevalence of anxiety was 9-fold higher in this study, compared with the general population of Kerala. While studies among people with diabetes report depression prevalence rates as high as 49% [13], the prevalence of CMDs in this study was likely to be lower because our study population group was only at risk of developing T2DM, and they had not yet encountered the added mental health strains associated with a diagnosis of diabetes and its accompanied self-management practices. The American Diabetes Association recommends regular depression screening among those with T2DM [56], but our findings suggest the need to enhance screening of mental health conditions in patients who are at risk of developing T2DM as well. Integrating mental health care into primary healthcare would improve early diagnosis, identifying at risk adults before CMDs could affect glycemic control and before the development of diabetes and any accompanying diabetes-related emotional stress could worsen anxiety and/or depressive symptoms.

Individuals who developed diabetes compared to those who did not, had significantly different clinical risk factor profiles, including higher central obesity, BMI, hypertension, and LDL cholesterol. However, there was no significant differences in mental health status at baseline between those who did and did not develop diabetes over the ensuing 24 months. This lack of association was confirmed by the GEE analyses, which showed that mental health at baseline was not related to diabetes incidence two years later. Although the evidence is mixed, this finding does not support a number of other previous studies, albeit those primarily undertaken in high-income countries [3, 6].

Given that diabetes has a prodromal period of three to six years [57, 58], it is possible that our null findings were due to the short follow-up of only 24 months. Or, that other clinical risk factors for diabetes have a greater and more direct impact on the development of diabetes. Previous studies examining the relationship between mental disorders and diabetes have also found that adjusting for additional diabetes risk factors produced lower risk estimates [3, 6]. Additionally, this is the first study in India to our knowledge that examines mental health outcomes in a community-based sample of people with a high risk of developing T2DM and one of very few undertaken in a LMIC. The unique socio-culture context of this population could help explain why we did not find a higher risk of developing diabetes among adults with moderate to severe depression and anxiety, as has been more frequently demonstrated in studies conducted in North America and Europe. A reflection of Asian collectivistic culture, the family plays a central role in Indian culture and has been shown to be a key resource in providing care for family members with mental disorders [59]. Recent research showed a significantly inverse relationship between social support and depression among people living with a chronic disease in India [60], demonstrating the potential for social support and mental health status to play intermediary roles in the relationship between environmental stressors and health behaviors that increase the risk of diabetes.

### Strengths and limitations

The strength of this study is its use of a large, randomized, community-based sample, which offers an examination of the relationship between CMDs and diabetes in a representative sample of people at risk for developing diabetes. Although data were obtained from a cluster randomized control trial, clustering was controlled for in all analyses and study participants were treated as a cohort while controlling for intervention effects in the GEE models. The short study timeline of two years limited the number of incident diabetes cases. A 7-year follow-up of the K-DPP trial is currently underway and the results obtained from those future studies will be able to build on the analysis conducted in this paper. Additionally, while the PHQ-9 and GAD-7 questionnaires are screening tools based on diagnostic criteria from the Diagnostic and Statistical Manual of Mental Disorders, additional clinical examinations are necessary to provide formal diagnosis. Finally, while the follow-up rate was high (95.8%), participants with one or more follow-up visits were different in some characteristics from those lost to follow-up. However, it is less likely that our results would be altered had these lost to follow-up individuals participated in the trial, as our main results were robust to those from the MI results based on complete datasets.

## Conclusion

This study documented higher prevalences of depression and anxiety than previously reported for the general population samples in Kerala, India, as well as those reported in WHO global mental health estimates. Despite this, these findings indicate that the presence of moderate to severe depression and anxiety symptoms was not associated with an increase in the risk of developing T2DM over a two-year period among high-risk individuals in this study. Longer follow-up needs to be undertaken in order to determine whether this relationship exists in this population, and the potential causal relationship is warranted for additional validation under the Mendelian Randomization framework [61–64].

## Supporting information

S1 Dataset Dataset for CCA. (DTA)

S2 Dataset Data analysis codes. (DO)

S3 Dataset Dataset for MI. (DTA)

S1 Table Multivariable analysis on MI datasets. (DOCX)

S2 Table Comparison of baseline characteristics. (DOCX)
