# Supplementary material for: The relationship between common mental disorders and incident diabetes among participants in the Kerala Diabetes Prevention Program (K-DPP)
Source: PLoS One. 2021 Jul 23;16(7):e0255217. doi: 10.1371/journal.pone.0255217 (PMC8301665; doi:10.1371/journal.pone.0255217)
Supplement: S1 Table — (DOCX) [file pone.0255217.s004.docx]

**S1 Table. Multivariable Analysis with Diabetes Incidence as the Dependent Variable on MI Datasets**

| **Model** | **Risk Factor** | **Odds Ratio** | **95% CI** | **P-value** |
| --- | --- | --- | --- | --- |
| Model 1^1^ | PHQ-9 Score ≥10  GAD-7 Score ≥10 | 1.04  0.69 | (0.54, 2.02)  (0.23, 2.05) | 0.90  0.50 |
| Model 2^2^ | PHQ-9 Score ≥10  GAD-7 Score ≥10 | 0.99  0.68 | (0.49, 1.97)  (0.23, 1.98) | 0.97  0.48 |
| Model 3^3^ | PHQ-9 Score ≥10  GAD-7 Score ≥10 | 1.05  0.69 | (0.52, 2.10)  (0.23, 2.09) | 0.89  0.52 |

PHQ, Patient Health Questionnaire; GAD-7, Generalized Anxiety Disorder-7; CI, confidence interval; MI, multiple imputation.

Odds ratios (and 95% CIs) were estimated using Generalized Estimating Equations with an exchangeable working matrix. Binomial family and logit link function were specified in the models. Standard errors were based on Huber-White sandwich estimator.

^1^Model 1: adjusted for study arm, sex, age, years in school, marital status, and family history of diabetes.

^2^Model 2: adjusted for study arm, sex, age, years in school, marital status, and family history of diabetes, alcohol use, tobacco use, leisure time physical activity, and fruit and vegetable consumption.

^3^Model 3: adjusted for study arm, sex, age, years in school, marital status, and family history of diabetes, alcohol use, tobacco use, leisure time physical activity, fruit and vegetable consumption, central obesity, hypertension, and LDL cholesterol.
