# Supplementary material for: The relationship between common mental disorders and incident diabetes among participants in the Kerala Diabetes Prevention Program (K-DPP)
Source: PLoS One. 2021 Jul 23;16(7):e0255217. doi: 10.1371/journal.pone.0255217 (PMC8301665; doi:10.1371/journal.pone.0255217)
Supplement: S2 Table — (DOCX) [file pone.0255217.s005.docx]

**S2 Table. Comparison of Baseline Characteristics between those Followed-up and those Lost to Follow-up**

| **Characteristics** | **Follow-up** | |
| --- | --- | --- |
|  | Yes  (N = 958) | No  (N = 49) |
| Age, in years; M (SD) | 46.0 (7.5) | 46.5 (7.1) |
| Sex, female; N (%) | 467 (48.8) | 8 (16.3) |
| Marital status, married; N (%) | 910 (95.0) | 48 (98.0) |
| Education, years completed; M (SD) | 9.7 (3.8) | 9.0 (3.1) |
| Family history of diabetes, Yes; *N* (%) | 499 (52.1) | 25 (51.0) |
| Alcohol use, Yes; N (%) | 196 (20.5) | 15 (30.6) |
| Tobacco use, Yes; N (%) | 181 (18.9) | 13 (26.5) |
| Fruit and vegetable intake, <5 servings per day; N (%) | 661 (69.0) | 35 (71.4) |
| Leisure time physical activity, No; N (%) | 762 (79.5) | 36 (73.5) |
| Sleep hours in the night, ≥7; N (%) | 528 (55.1) | 30 (61.2) |
| Central obesity, Yes; N (%) | 667 (69.8) | 32 (65.3) |
| Hypertension, Yes; N (%) | 211 (22.0) | 14 (28.6) |
| LDL cholesterol, in mg/dl; M (SD) | 147.9 (35.7) | 145.5 (37.9) |
| PHQ-9, N (%)             <10             ≥10 | 855 (92.5)  69 (7.5) | 43 (91.5)  4 (8.5) |
| GAD-7, N (%)             <10             ≥10 | 901 (94.4)  53 (5.6) | 47 (95.9)  2 (4.1) |

M, mean; SD, standard deviation; LDL, low density lipoprotein; PHQ, Patient Health Questionnaire; GAD-7, Generalized Anxiety Disorder-7. Percentages may not add up to 100% because of rounding.
